# Association between personality characteristics and sleep quality among Chinese middle-aged and older adults: evidence from China family panel studies

**DOI:** 10.1186/s12889-023-17352-6

**Published:** 2023-12-05

**Authors:** Zhen Wang, Zhi Zeng

**Affiliations:** 1 School of Public Health, Hubei University of Medicine, Hubei, China; 2 Center of Health Administration and Development Studies, Hubei University of Medicine, Hubei, China

**Keywords:** Personality characteristics, Sleep duration, Sleep perception, Middle-aged and older adults, China

## Abstract

**Background:**

Poor sleep quality will have adverse effects on physical and mental health, quality of life and other aspects of middle-aged and older adults. Sleep quality is affected by many factors. Whether the sleep quality measures of the participants had changed in the previous or subsequent time period is not easily taken into account. Moreover, there have been no studies on this topic in Chinese middle-aged and older adults. The objective of this study was to mitigate the bias of sleep quality assessment, and analyze the association between personality traits and sleep quality in Chinese middle-aged and older adults.

**Methods:**

The data came from the China Family Panel Studies (CFPS). A total of 6031 participants aged ≥ 45 years were included in this study. Personality characteristics were evaluated based on the scores of each dimension of Big Five personality traits. Sleep duration and sleep perception were used as indicators to measure sleep quality. Logistic models were used to analyze the relationship between personality traits and sleep duration or sleep perception, respectively.

**Results:**

4.5% of the participants had abnormal sleep duration, and 14.4% had a pessimistic sleep perception. Conscientiousness was rated the highest among the personality traits (3.97 ± 0.6). Participants with higher scores for extraversion personality traits had more normal sleep duration (OR = 0.77, 95% CI: 0.64–0.93) and more optimistic sleep perception (OR = 0.86, 95% CI: 0.76–0.96). Using the Internet and feeling unwell in the past week have a moderating effect on the impact of conscientiousness personality characteristics on sleep duration or sleep perception, respectively (but not overall sleep quality). In addition, participants with a spouse or no recent physical discomfort tended to have a normal sleep duration and a more optimistic sleep perception.

**Conclusions:**

The higher the score of extraversion personality traits, the better the overall sleep quality of middle-aged and older adults. Having a spouse and feeling unwell were the important factors affecting their sleep quality. Specific personality traits intervention should be carried out for middle-aged and older adults with poor sleep quality to make their personality traits are closer to extraversion. In addition, middle-aged and older adults without spouses should be encouraged to marry or remarry. We will strengthen health management and medical expenditures for middle-aged and older adults.

## Introduction

Poor sleep quality is a widespread and significant public health problem. The reported prevalence of sleep problems (such as insomnia) in the United States increased from 17.5%(about 37.5 million adults) in 2002 to 19.2%(over 46.2 million adults) in 2012 [1]. Sleep quality plays an important role in maintaining physical and mental health [2], and contributes positively to excellent performance of daily activities [3, 4]. Poor sleep is strongly associated with the development of several diseases, including obesity [5], depression [6, 7], poor job performance [8–10], unethical and high-risk behaviors [11–13], low life satisfaction and well-being [14], cognitive decline and dementia [15, 16], and increased risk of stroke, including ischemic and hemorrhagic strokes [17, 18]. In addition, sleep patterns tend to change with age, for example, total sleep duration decreases in older adults [19], which has the potential to have clear adverse effects on their people’s physical and mental health [20], as well as on their daytime functioning and quality of life [3].

In the existing research, there exists a widely accepted and adopted personality classification method, namely the “Big Five” personality classification method, which divides personality traits into five main dimensions, namely conscientiousness, extraversion, openness, neuroticism and agreeableness [21]. Each dimension is linear, meaning that individuals have a position on a continuum [22]. Conscientiousness refers to gratuitous behavior, with lack of unreliability and persistence at one end of the continuum as well as diligence, consistency and planning at the other end [23]. Extraversion is related to the degrees of energy, sociability and optimism and the quality of intrapersonal interaction [24]. Openness to new experiences refers to the extent to which a person uses his/her creativity and imagination in everyday life [25]. Neuroticism is linked to psychological adjustment and negative emotions such as sadness [26]. Finally, individuals with high agreeableness tend to be modest, polite, altruistic and trustworthy [27].

Some studies have shown that personality traits have an effect on sleep. As for conscientiousness, a study of the sleep status of American adults found that conscientiousness predicted longer sleep duration [28]. There was also a study on the sleep status of oncology nurses that confirmed that conscientiousness was significantly positively associated with adequate sleep and lower insomnia symptoms [29]. Another study of sleep in Italian adults found that conscientiousness was protective of sleep quality [30]. Regarding extraversion, a study of American adults found that extraversion was associated with shorter sleep duration [28]. In terms of openness, a study of sleep quality among Black adults in Florida showed that individuals with low openness (e.g., aesthetics and ideas) were more likely to report poor sleep [31]. In regard to neuroticism, a study of sleep in a group of American college students found that college students with neuroticism personality traits tended to have poorer sleep and insomnia symptoms [32]. In addition, adults with higher scores of neuroticism personality traits are more likely to have trouble falling asleep and to fall asleep later at night, and thus are more likely to have a poor sleep quality [33, 34]. At the level of agreeableness, a study of sleep in Australian adults found that those with higher agreeableness scores slept longer [33]. In addition, agreeableness personality traits were significantly positively correlated with adequate sleep and lower insomnia symptoms [29]. It can be concluded that there is a certain association between personality traits and sleep quality. However, most of the previous studies focus on the adult group, while there are relatively few studies on the middle-aged and older groups. Moreover, some studies included sleep quality indicators of participants without considering whether they had changed in the previous or the subsequent period.

With the explosive spread of the Internet and mobile phones, people’s normal work and rest time has been significantly affected [35]. One study reported that Internet use in older adults was negatively associated with later going to bed, waking up earlier, and sleep duration, but not with sleep quality. Their use of the Internet may keep them awake, but it does not affect their mental health [36]. There is also a study that shows a significant association between Internet use and sleep quality [37]. In addition, a study involving personality characteristics found that Internet addiction of college students was positively correlated with neuroticism of the Big Five, but negatively correlated with conscientiousness of the Big Five [38]. This raises a question: Does Internet use have a moderating effect on the relationship between personality traits and sleep quality?

Therefore, this study will fill these research gaps and provide a new perspective and basis for the research in the field of personality and sleep quality. The purpose of this study was to analyze the association between personality characteristics and sleep quality in middle-aged and older Chinese. This will have certain academic value and significance for enriching theoretical research in related fields, understanding sleep problems in middle-aged and older adults, and formulating intervention measures and policy guidance for sleep health in middle-aged and older adults.

## Materials and methods

### Data and samples

The data of this study comes from the China Family Panel Studies (CFPS) [39], which is implemented by the Institute of Social Science Survey of Peking University. It aims to track and collect data at three levels of individuals, families and communities, reflect the social changes in China from the perspectives of population, economy, health and education, and provide data basis for academic research and public policy formulation. CFPS launched the first round of baseline survey in 2010. The baseline sample covered 25 provinces (autonomous regions and municipalities), involving 14,960 households and 42,590 individuals. They were followed up every two years, and there have been six waves of national surveys so far. 2020 CFPS was released in 2023.

In the 2020 CFPS survey, 12,735 participants were adults aged ≥ 45 years. In order to make the results robust, we narrowed the scope of participants to: The survey results of sleep duration and sleep perception in the previous wave (2018) are consistent with those in the current wave (2020), that is, both consecutive waves are normal or abnormal (for example, a person’s sleep duration is uniformly normal or abnormal in the two waves, and his sleep perception is also uniformly normal or abnormal in the two waves). It could effectively avoid biased results due to recent unexpected events affecting their sleep quality. After removing outliers and missing values of relevant variables, we ended up including 6031 participants in the study.

### Assessment of personality traits (Independent variable)

This paper uses the “Big Five” personality classification method widely used in academia to assess the variables of personality traits of the participants. In the CFPS, there are questionnaires that specifically address the personality traits of adult respondents. Based on the theoretical framework of the widely used “Big Five” personality measurement tool Neo-Pi-R, and on the basis of the Chinese adjective “Big Five” personality scale (BFFP-CAS), this questionnaire constructs a five-dimensional investor personality trait scale based on the CFPS questionnaire, covering conscientiousness, extraversion, openness, neuroticism and agreeableness [40]. In this study, the average score of each dimension of the “Big Five” Personality Inventory (BFFP-CAS) was calculated to evaluate the personality characteristics of participants.

### Measurement of sleep quality (dependent variable)

The indicators of sleep quality in this study include sleep duration and sleep perception.

Sleep duration is an important objective index reflecting individual sleep quality, and also an indirect or direct factor affecting individual health status. For example, long-term short sleep duration can induce different degrees of related diseases, such as cardiovascular diseases and mental diseases [41]. The dependent variable “sleep duration” in this study is sorted out from the questions about night sleep time in the CFPS questionnaire, such as “how many hours do you sleep every day in general,“ “how many hours do you sleep every day on working days,“ and “how many hours do you sleep every day on rest days.“ Based on the research on abnormal sleep duration in middle-aged and older adults [42, 43], this study defined insufficient sleep (≤ 4 h) and excessive sleep (≥ 10 h) as abnormal sleep duration, and assigned a value of 1; Sleep duration between 4 and 10 h (excluding 4 and 10 h) was defined as normal sleep duration and assigned a value of 0.

Sleep perception is an individual’s self-subjective evaluation of their own sleep status, which usually includes the evaluation of sleep efficiency and effect, and is an important subjective index reflecting individual sleep quality. According to the relevant diagnostic criteria of sleep disorders in the International Classification of Sleep Disorders (Third edition) edited by the American Academy of Sleep Medicine [44] and the Sleep Quality Index scale of the University of Pittsburgh [45], subjective sleep perception and objective sleep duration are important reference indicators to judge sleep quality. The dependent variable “sleep perception” in this study is organized from the question “I feel bad sleep” in the CFPS questionnaire, which adopts the scoring method from 1 to 4, with scores of 1 and 2 indicating good feeling and scores of 3 and 4 indicating poor feeling. In this paper, scores 3 and 4 were defined as pessimistic perception and assigned a value of 1. Scores 1 and 2 were defined as optimistic perception and assigned a value of 0.

### Control variables

The control variables in this study include: Gender (male and female), age group (45–59 years, 60–74 years and ≥ 75 years), residence (urban and rural), having a spouse (yes or no), education (illiterate, primary school, junior high school and above), feeling unwell in the past week (yes or no), smoking in the past month (yes or no), drinking no less than three times a week in the past month (yes or no), doing physical exercise (yes or no), having a job (yes or no), and using the Internet (yes or no).

### Statistic analysis

Continuous variables are expressed as means (standard deviation, SD) and categorical variables as numbers (percentages). Differences between the two groups were compared using chi-square test or t-test. Results with *P* values < 0.05 were considered statistically significant. Logistic regression model was used to analyze the association between personality characteristics and sleep duration or sleep perception in middle-aged and older adults. All statistical analyses were performed by Stata16.0.

## Results

### Sample characteristics

Table 1 shows the demographic characteristics of the participants. Of the 6,031 participants, 52.6% were male, more than half (57.4%) were 45–59 years old, and nearly two-thirds (67.9%) lived in rural areas. The vast majority (89.6%) of participants had a spouse. About half (51.6%) have attained Junior high school education or above. 29.3% felt unwell in the past week. 29.2% smoked and 16.9% drank. About two-thirds (67.4%) did not exercise. 62.6% of the participants had a job. Less than half (45.4%) used the Internet. 4.5% of the participants had abnormal sleep duration, and 14.4% had a pessimistic sleep perception. The mean scores of participants on the five dimensions of personality traits (conscientiousness, extraversion, openness, neuroticism, and agreeableness) were 3.97 (SD = 0.6), 3.38 (SD = 0.7), 2.89 (SD = 0.8), 3.90 (SD = 0.6), and 3.13 (SD = 0.9).

**Table 1:** Characteristics of participants according to cluster of sleep duration /sleep perception

		Total No (%)	Sleep duration		*P*-value from Chi−2 / t-test	Sleep perception		*P*-value from Chi−2 / t-test
			Normal (95.5%)	Abnormal (4.5%)		Optimistic (85.6%)	Pessimistic (14.4%)	
			No (%)	No (%)		No (%)	No (%)	
Gender	Male	3171 (52.6)	3028 (52.6)	143 (53.0)	0.897	2888 (56.0)	283 (32.5)	<0.001
	Female	2860 (47.4)	2733 (47.4)	127 (47.0)		2272 (44.0)	588 (67.5)	
Age group	45–59 years	3464 (57.4)	3330 (57.8)	134 (49.3)	<0.001	3002 (58.2)	462 (53.0)	0.018
	60−74years	2202 (36.5)	2099 (36.4)	103 (38.2)		1851 (35.9)	351 (40.3)	
	≥ 75 years	365 (6.1)	332 (5.8)	33 (12.2)		307 (6.0)	58 (6.7)	
Residence	Urban	1938 (32.1)	1884 (32.7)	54 (20.0)	<0.001	1690 (32.8)	248 (28.5)	0.012
	Rural	4093 (67.9)	3877 (67.3)	216 (80.0)		3470 (67.3)	623 (71.5)	
Having a spouse	No	629 (10.4)	583 (10.1)	46 (17.0)	<0.001	497 (9.6)	132 (15.2)	<0.001
	Yes	5402 (89.6)	5178 (89.9)	224 (83.0)		4663 (90.4)	84.8 (84.9)	
Education	Illiteracy	1491 (24.7)	1394 (24.2)	97 (35.9)	<0.001	1202 (23.3)	289 (33.2)	<0.001
	Primary	1428 (23.7)	1365 (23.7)	63 (23.3)		1217 (23.6)	211 (24.2)	
	Junior high school and above	3112 (51.6)	3002 (52.1)	110 (40.7)		2741 (53.1)	371 (42.6)	
Feeling unwell	No	4263 (70.7)	4098 (71.1)	165 (61.1)	<0.001	3848 (74.6)	415 (47.7)	<0.001
	Yes	1768 (29.3)	1663 (28.9)	105 (38.9)		1312 (25.4)	456 (52.4)	
Smoking	No	4271 (70.8)	4089 (71.0)	182 (67.4)	0.207	3571 (69.2)	700 (80.4)	<0.001
	Yes	1760 (29.2)	1672 (29.0)	88 (32.6)		1589 (30.8)	171 (19.6)	
Drinking	No	5013 (83.1)	4787 (83.1)	226 (83.7)	0.794	4257 (82.5)	756 (86.8)	0.002
	Yes	1018 (16.9)	974 (16.9)	44 (16.3)		903 (17.5)	115 (13.2)	
Doing physical exercise	No	4065 (67.4)	3862 (67.0)	203 (75.2)	0.005	3468 (67.2)	597 (68.5)	0.438
	Yes	1966 (32.6)	1899 (32.96)	67 (24.8)		1692 (32.8)	274 (31.5)	
Having a job	No	2258 (37.4)	2152 (37.4)	106 (39.3)	0.527	1846 (35.8)	412 (47.3)	<0.001
	Yes	3773 (62.6)	3609 (62.7)	164 (60.7)		3314 (64.2)	459 (52.7)	
Using the Internet	No	3293 (54.6)	3102 (53.8)	191 (70.7)	<0.001	2763 (53.6)	530 (60.9)	<0.001
	Yes	2738 (45.4)	2659 (46.2)	79 (29.3)		2397 (46.5)	341 (39.2)	
Big five personality								
Conscientiousness	Mean (SD)	3.97 (0.6)	3.97 (0.6)	3.96 (0.6)	0.660	3.99 (0.6)	3.89 (0.6)	<0.001
Extraversion	Mean (SD)	3.38 (0.7)	3.39 (0.7)	3.30 (0.7)	0.042	3.40 (0.7)	3.28 (0.7)	<0.001
Openness	Mean (SD)	2.89 (0.8)	2.88 (0.8)	2.97 (0.7)	0.072	2.83 (0.8)	3.25 (0.7)	<0.001
Neuroticism	Mean (SD)	3.90 (0.6)	3.90 (0.6)	3.86 (0.6)	0.268	3.91 (0.6)	3.83 (0.6)	<0.001
Agreeableness	Mean (SD)	3.13 (0.9)	3.12 (0.9)	3.21 (0.9)	0.105	3.14 (0.9)	3.06 (0.9)	0.011

### Univariate analysis

Table 1 shows the results of comparing the difference between the two groups with normal sleep duration and abnormal sleep duration. There were significant differences in age (*p* < 0.001), residence (*p* < 0.001), having a spouse (*p* < 0.001), education (*p* < 0.001), feeling unwell (*p* < 0.001), doing physical exercise (*p* < 0.01), using the Internet (*p* < 0.001) and extraversion (*p* < 0.05) between the two groups.

In addition, from Table 1, we can also see the results of comparing the differences between the two groups of optimistic and pessimistic sleep perception. There were significant differences in gender (*p* < 0.001), age (*p* < 0.05), residence (*p* < 0.01), having a spouse (*p* < 0.001), education (*p* < 0.001), feeling unwell (*p* < 0.001), smoking (*p* < 0.001), drinking (*p* < 0.001), having a job (*p* < 0.001), using the Internet (*p* < 0.001), and the dimensions of Big Five (*p* < 0.05 or 0.001).

### Association between personality traits and sleep duration

**Fig. 1:**
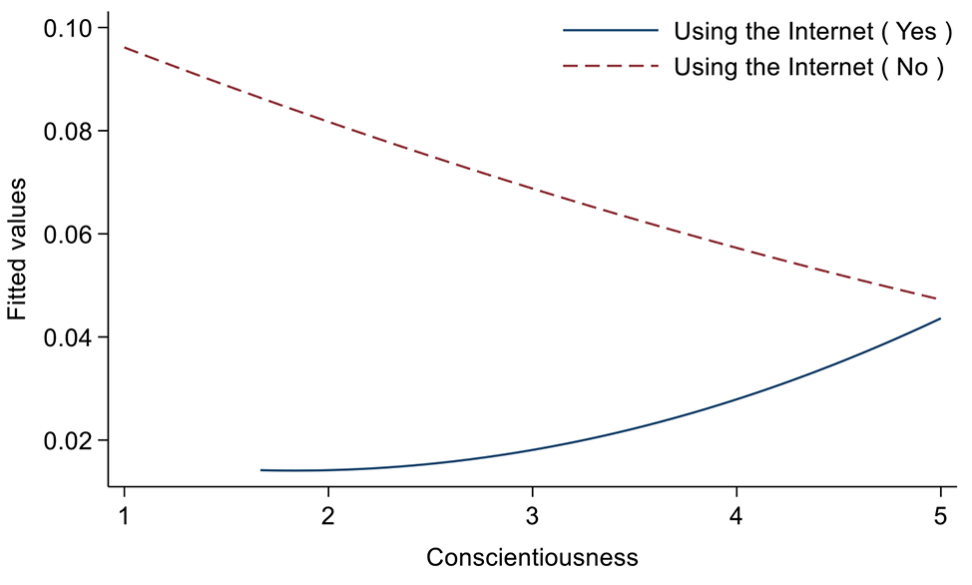
Moderating effect diagram of “Using the Internet”

Table 2 shows the association between personality traits and sleep duration. We only included control variables in Model 1, and then included independent variables in Model 2, and further included interaction terms in Model 3. In Model 2, extraversion (OR = 0.77, 95% CI: 0.64–0.93) and agreeableness (OR = 1.21, 95% CI: 1.04–1.40) were both significant factors for abnormal sleep duration. Specifically, participants with higher scores on extraversion tended to sleep more normally. Participants with higher agreeableness scores tended to have more abnormal sleep duration. In addition, the interaction term “Conscientiousness×Using the Internet” had a significant interaction effect (OR = 1.81, 95% CI: 1.15–2.85). Figure 1 is a diagram of the interaction effect, in which the closer the value of the vertical axis was to 0, the more normal the sleep duration was. For participants who used the Internet, the higher their scores on the conscientiousness personality trait, the more their sleep duration tended to be abnormal. For participants who did not use the Internet, the higher their scores on the conscientiousness personality trait, the more normal their sleep duration tended to be. But overall, those who used the Internet had more normal sleep durations than those who didn’t, regardless of their ratings of conscientiousness personality traits. For the control variables, participants aged ≥ 75 years, living in rural areas, not having a spouse, feeling unwell, or not using the Internet were more likely to have abnormal sleep duration (*p* < 0.05 or 0.01).

**Table 2:** Associations between Big Five personalities and sleep duration

		Model 1		Model 2		Model 3	
		OR	95% CI	OR	95% CI	OR	95% CI
Gender	Male	Ref.		Ref.		Ref.	
	Female	0.95	[0.69,1.31]	0.98	[0.71,1.35]	0.98	[0.71,1.36]
Age group	45–59 years	Ref.		Ref.		Ref.	
	60−74years	1.02	[0.76,1.37]	1.04	[0.78,1.39]	1.04	[0.78,1.40]
	≥ 75 years	1.85**	[1.16,2.95]	1.92**	[1.20,3.06]	1.92**	[1.20,3.05]
Residence	Urban	Ref.		Ref.		Ref.	
	Rural	1.66**	[1.18,2.35]	1.67**	[1.18,2.37]	1.67**	[1.18,2.36]
Having a spouse	No	Ref.		Ref.		Ref.	
	Yes	0.66*	[0.46,0.93]	0.66*	[0.47,0.94]	0.67*	[0.47,0.95]
Education	Illiteracy	Ref.		Ref.		Ref.	
	Primary	0.77	[0.55,1.08]	0.79	[0.56,1.10]	0.79	[0.56,1.11]
	Junior high school and above	0.85	[0.61,1.18]	0.86	[0.62,1.20]	0.86	[0.62,1.20]
Feeling unwell	No	Ref.		Ref.		Ref.	
	Yes	1.46**	[1.13,1.89]	1.44**	[1.11,1.87]	1.44**	[1.11,1.86]
Smoking	No	Ref.		Ref.		Ref.	
	Yes	1.23	[0.89,1.70]	1.25	[0.90,1.72]	1.25	[0.91,1.73]
Drinking	No	Ref.		Ref.		Ref.	
	Yes	0.93	[0.65,1.33]	0.93	[0.65,1.33]	0.94	[0.66,1.34]
Doing physical exercise	No	Ref.		Ref.		Ref.	
	Yes	0.88	[0.65,1.18]	0.88	[0.65,1.19]	0.88	[0.65,1.18]
Having a job	No	Ref.		Ref.		Ref.	
	Yes	0.96	[0.72,1.28]	0.93	[0.70,1.25]	0.94	[0.70,1.26]
Using the Internet	No	Ref.		Ref.		Ref.	
	Yes	0.64**	[0.47,0.87]	0.62**	[0.46,0.85]	0.06**	[0.01,0.37]
Big five personality							
Conscientiousness				1.02	[0.83,1.26]	0.88	[0.69,1.11]
Extraversion				0.77**	[0.64,0.93]	0.78**	[0.65,0.93]
Openness				1.01	[0.85,1.21]	1.02	[0.85,1.21]
Neuroticism				0.92	[0.74,1.15]	0.92	[0.74,1.14]
Agreeableness				1.21*	[1.04,1.40]	1.20*	[1.03,1.39]
Interaction item							
Conscientiousness×Using the Internet						1.81**	[1.15,2.85]
Constant		0.05***	[0.03,0.10]	0.08***	[0.02,0.33]	0.15**	[0.04,0.62]
Pseudo R^2^		0.03		0.04		0.04	
Observation		6031		6031		6031	

*p < 0.05, **p < 0.01,***p < 0.001

### Association between personality traits and sleep perception

**Fig. 2:**
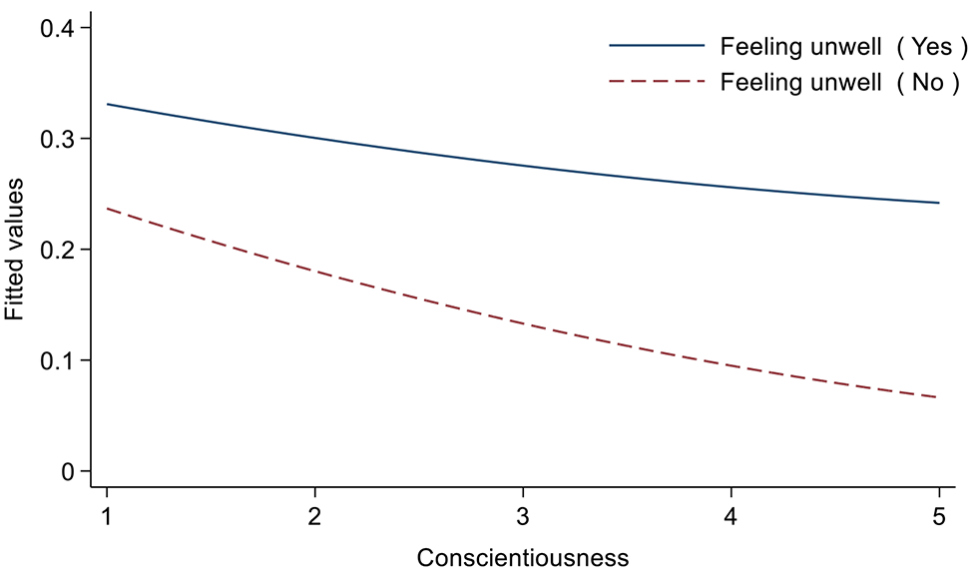
Moderating effect diagram of “Feeling unwell”

Table 3 shows the association between personality traits and sleep perception. We only included control variables in Model 1, and then included independent variables in Model 2, and further included interaction terms in Model 3. In Model 2, extraversion (OR = 0.86, 95% CI: 0.76–0.96), openness (OR = 1.91, 95% CI: 1.70–2.14) and neuroticism (OR = 0.86, 95% CI: 0.76–0.99) were significant factors for abnormal sleep perception (*p* < 0.05, 0.01 or 0.001). Specifically, participants with higher extraverted personality scores tended to have sleep perceptions that tended to be more optimistic. Participants with higher scores on openness tended to have more pessimistic sleep perceptions. Participants with higher scores on neuroticism tended to have more optimistic sleep perceptions. In addition, the interaction term “Conscientiousness×Feeling unwell” had a significant interaction effect (OR = 1.31, 95% CI: 1.04–1.66). Figure 2 is a diagram of the interaction effect, in which the closer the value of the vertical axis was to 0, the more optimistic the sleep perception was. Regardless of whether the participants felt physically ill or not, the higher the score of conscientiousness personality trait was, the more optimistic their sleep perception was. But overall, sleep perceptions without physical discomfort tended to be more optimistic than those with physical discomfort. For the control variables, participants who were female, did not have a spouse, felt ill, drank alcohol, or did not have a job were more likely to have pessimistic sleep perception (*p* < 0.05, 0.01 or 0.001).

**Table 3:** Associations between Big Five personalities and sleep perception

		Model 1		Model 2		Model 3	
		OR	95% CI	OR	95% CI	OR	95% CI
Gender	Male	Ref.		Ref.		Ref.	
	Female	2.33***	[1.90,2.86]	2.12***	[1.71,2.62]	2.13***	[1.73,2.63]
Age group	45–59 years	Ref.		Ref.		Ref.	
	60−74years	1.14	[0.87,1.24]	1.14	[0.95,1.36]	1.14	[0.95,1.36]
	≥ 75 years	1.06	[0.65,1.28]	1.06	[0.74,1.50]	1.06	[0.74,1.50]
Residence	Urban	Ref.		Ref.		Ref.	
	Rural	1.13	[1.00,1.47]	1.13	[0.93,1.38]	1.13	[0.93,1.38]
Having a spouse	No	Ref.		Ref.		Ref.	
	Yes	0.74**	[0.59,0.92]	0.76*	[0.60,0.95]	0.76*	[0.60,0.95]
Education	Illiteracy	Ref.		Ref.		Ref.	
	Primary	0.94	[0.76,1.15]	0.98	[0.79,1.21]	0.98	[0.79,1.21]
	Junior high school and above	0.86	[0.70,1.06]	0.94	[0.76,1.16]	0.94	[0.76,1.16]
Feeling unwell	No	Ref.		Ref.		Ref.	
	Yes	2.87***	[2.46,3.33]	2.66***	[2.28,3.10]	0.92	[0.36,2.31]
Smoking	No	Ref.		Ref.		Ref.	
	Yes	0.99	[0.79,1.24]	1.00	[0.79,1.26]	1.00	[0.80,1.27]
Drinking	No	Ref.		Ref.		Ref.	
	Yes	1.30*	[1.03,1.65]	1.34*	[1.05,1.70]	1.34*	[1.05,1.70]
Doing physical exercise	No	Ref.		Ref.		Ref.	
	Yes	1.03	[0.87,1.23]	1.07	[0.90,1.27]	1.07	[0.90,1.28]
Having a job	No	Ref.		Ref.		Ref.	
	Yes	0.72***	[0.61,0.86]	0.72***	[0.60,0.85]	0.72***	[0.60,0.85]
Using the Internet	No	Ref.		Ref.		Ref.	
	Yes	0.90	[0.75,1.08]	0.92	[0.76,1.10]	0.92	[0.77,1.10]
Big five personality							
Conscientiousness				0.89	[0.78,1.01]	0.78**	[0.65,0.93]
Extraversion				0.86**	[0.76,0.96]	0.86**	[0.76,0.96]
Openness				1.91***	[1.70,2.14]	1.90***	[1.70,2.13]
Neuroticism				0.86*	[0.76,0.99]	0.86*	[0.75,0.99]
Agreeableness				0.99	[0.90,1.08]	0.99	[0.90,1.08]
Interaction item							
Conscientiousness×Feeling unwell						1.31*	[1.04,1.66]
Constant		0.10***	[0.07,0.16]	0.07***	[0.03,0.17]	0.12**	[0.05,0.32]
Pseudo R^2^		0.08		0.12		0.12	
Observation		6031		6031		6031	

*p < 0.05, **p < 0.01,***p < 0.001

## Discussion

Using nationally representative CFPS data, this study aims to evaluate the impact of personality characteristics on sleep quality measured by sleep duration and sleep perception in middle-aged and older Chinese. The results of this study have implications for clinical practice guidance as well as health policy formulation.

In this study, the Big Five personality (conscientiousness, extraversion, openness, neuroticism and agreeableness) was used to measure the personality characteristics of middle-aged and older adults in China, and sleep duration and sleep perception were used to measure sleep quality, so as to analyze the relationship between personality characteristics and sleep quality. The results of this study showed that middle-aged and older adults with higher scores of extraversion personality traits had better sleep quality, that is, they had both normal sleep duration and optimistic sleep perception. Some scholars have conducted similar studies on other groups of people, such as adults aged 18–45 years old, and found that higher scores of extraversion personality are associated with better sleep continuity [46]. Some scholars have also studied the sleep of Indian college students and found that college students with extraversion and conscientiousness have relatively good sleep conditions [47]. All these are roughly consistent with the direction of the conclusions of this study. Middle-aged and older adults with higher scores of extraversion personality traits have better sleep quality, and the possible reasons are analyzed as follows: first, social support is considered to be one of the important factors to promote health and enhance sleep quality [48]. Extroverts prefer to interact with others, and they generally have wider social circles and more opportunities to socialize, which may provide more social support and emotional support, thus favoring having a good night’s sleep. Second, mood has been shown to be an important factor in sleep quality [49, 50]. Extroverts, who typically have positive and optimistic emotions and a high degree of emotional stability, are more inclined to experience positive emotions such as happiness, excitement, and satisfaction. A positive emotional state can reduce stress and anxiety, promote physical and mental relaxation, and help improve sleep quality. Third, exercise is considered to be an important factor affecting sleep quality [51]. Extroverts are generally more proactive and enjoy participating in various activities and sports. Their active engagement in physical and mental activities may help burn energy, reduce tension, and promote sleep.

The results of this study suggest that middle-aged and older adults with high agreeableness are more likely to have an abnormal sleep duration. In two large samples of Australian and Finnish adults, high agreeableness was associated with abnormal sleep duration [33], consistent with the results of this study. The possible reason: although people with higher agreeableness generally have an optimistic and positive mindset, they tend to be less satisfied with their lives [52]. As a result, the psychological burden of facing the stresses and challenges in their lives may have a negative impact on sleep. In addition, they often put more emphasis on social interactions and relationships [53], which can affect sleep duration [54]. This study also found that middle-aged and older adults with high openness were more likely to have a pessimistic sleep perception. A study of sleep quality in Australian adults found that higher levels of openness were associated with poorer sleep quality [55]. In general, openness leads to positive outcomes such as resilience, lower stress, and improved mental and physical health [56]. However, some of the outcomes associated with openness (such as greater intelligence and study/work engagement) may be associated with lower quality sleep. Higher intelligence is associated with poorer sleep, possibly because high intelligence individuals exhibit greater rumination and cognitive activation [57]. This study showed that middle-aged and older adults with high neuroticism tend to be optimistic in terms of sleep perception. This is contrary to the usual findings [58]. It may be because they are 45 years old and above, with certain life experience and summary. As a result, they have more effective psychological adjustment strategies, are better able to cope with anxiety and mood swings, and thus feel more optimistic about sleep. Overall, these findings only suggest that personality traits (agreeableness, openness, and neuroticism) have significant effects on only one of the measures of sleep quality, and the effects on overall sleep quality need to be further studied and explored.

The interaction model in this paper shows that in the study of the relationship between conscientiousness and sleep duration, the variable “using the Internet” has a significantly negative moderating effect. But overall, those who used the Internet had more normal sleep durations than those who didn’t, regardless of their ratings of conscientiousness personality traits. However, previous studies have mostly found that Internet use is a risk factor for abnormal sleep duration [59]. This could be because people who have trouble sleeping have turned to the Internet to find ways to improve their sleep. Through online chat tools, they make up for the lack of spiritual comfort from children, friends or relatives in reality, and obtain emotional support, which has a positive impact on sleep. In the study on the relationship between conscientiousness and sleep perception, the variable “feeling unwell” has a significant moderating effect. The less unwell the participant felt and the higher the conscientiousness score was, the more optimistic their sleep perception was. However, these factors only have a moderating effect on the influence of a certain index. As for which factors have a moderating effect on the influence of personality characteristics on the overall sleep quality, further in-depth research and exploration are still needed.

This study shows that having a spouse and feeling unwell are significant factors affecting the sleep quality of middle-aged and older adults. Specifically, middle-aged and older adults with a spouse have relatively better sleep quality. Studies have shown that having a spouse is an important factor affecting the sleep quality of middle-aged and older adults [60]. The possible reasons are as follows: first, they can usually get more social support and emotional support. Emotional companionship and understanding from a spouse can alleviate the stress and anxiety that middle-aged and older adults may face in their daily lives, which may help reduce sleep problems and improve sleep quality. Second, they are more likely to have a regular routine. Spouses can supervise and manage the daily schedule of middle-aged and older adults and help them establish good sleep habits, such as a regular wake-up time and bedtime. This regularity can promote better sleep quality. Third, they may feel more secure and at ease, especially at night. Middle-aged and older adults are faced with a high sense of loneliness [61]. Having a spouse around can provide psychological security, reduce this sense of loneliness and anxiety, and thus improve sleep quality. In addition, middle-aged and older adults who did not feel unwell had relatively good sleep quality. The possible reasons are as follows: First, they may not have the underlying physical discomfort (such as pain, discomfort, etc.), which can reduce problems such as difficulty falling asleep, waking up during the night, and sleep interruption. Second, they may be more likely to follow a regular schedule because they don’t have physical discomfort that interferes with their sleep schedule. A regular sleep schedule helps maintain a stable circadian rhythm. Third, they may be psychologically more positive and optimistic and hold a more positive attitude towards things. This mental state may help reduce the interference of anxiety, depression and other negative emotions on sleep, thus improving sleep quality.

The strengths of this study are as follows: first, in order to make the research results robust, we selected participants whose sleep duration and sleep perception were consistent in the survey results of two consecutive waves (2018, 2020), that is, these two indicators were uniformly normal or abnormal in the two consecutive waves respectively. It effectively avoids the bias of the study results caused by the impact of recent emergencies on sleep quality. Second, the large sample size of this study can provide sufficient statistical power to make the results more representative and reliable. Third, in addition to the data on personality characteristics and sleep quality, other possible influencing factors such as marital status, education level, and health behaviors were also considered in this study, so as to more comprehensively analyze the factors affecting sleep quality in middle-aged and older adults.

There are some limitations to this study. First, the information related to sleep duration, sleep perception and personality characteristics in CFPS data is self-reported, and recall bias or subjective evaluation may have a certain impact on the research results. Second, the results of this study may be more applicable to middle-aged and older adults, but cannot be easily generalized to other people. Third, the perspectives considered in this study are limited, and factors such as living environment, social capital, and chronic diseases can be included in future studies. Fourth, this study adopts a cross-sectional design, which makes the conclusions of the study not causal.

## Conclusions and implications

Based on nationally representative data, we found that middle-aged and older adults with higher scores of extraverted personality traits had better overall sleep quality, as reflected by more normal sleep duration and optimistic sleep perception. In addition, having a spouse and feeling unwell are also important factors affecting the sleep quality of middle-aged and older adults.

Based on the research conclusions, we have the following policy implications. First, personality traits are stable and hard to change. Therefore, on the premise of accepting middle-aged and older adults as they are, effective sleep quality intervention measures should be provided according to their tendencies, such as sleep health education and relaxation training therapy for middle-aged and older adults with poor sleep quality who are less inclined to extroversion. Second, Encourage the establishment of clubs with similar age groups to better provide emotional support according to the environment of middle-aged and older adults. In addition, for middle-aged and older adults without spouses who intend to marry, the government can set up special institutions or departments to provide relevant information and support, such as providing marriage counseling and psychological support. Third, health management and medical support for middle-aged and older adults should be strengthened to help them deal with physical discomfort. For example, a system of health records and regular physical examinations can be established to detect and treat potential health problems in a timely manner. Actively carry out health education and promotion activities to prevent or alleviate health problems.

## Data Availability

The data from the China Family Panel Studies (CFPS) is open to the public and can be obtained free of charge through this website (http://www.isss.pku.edu.cn/cfps/).

## Abbreviations

CFPS: China Family Panel Studies
OR: Odds Ratio
CI: Confidence Interval
